# Survival of *Escherichia coli* and *Listeria innocua* on Lettuce after Irrigation with Contaminated Water in a Temperate Climate

**DOI:** 10.3390/foods10092072

**Published:** 2021-09-02

**Authors:** Bernardino Machado-Moreira, Karl Richards, Florence Abram, Fiona Brennan, Michael Gaffney, Catherine M. Burgess

**Affiliations:** 1 Teagasc Food Research Centre Ashtown, D15 KN3K Dublin, Ireland; bernardinommoreira@gmail.com (B.M.-M.); michael.gaffney@teagasc.ie (M.G.); 2 School of Natural Sciences, National University of Ireland Galway, H91 TK33 Galway, Ireland; Florence.abram@nuigalway.ie; 3 Teagasc Johnstown Castle Environmental Research Centre, Y35 Y521 Wexford, Ireland; karl.richards@teagasc.ie (K.R.); fiona.brennan@teagasc.ie (F.B.)

**Keywords:** irrigation, *Listeria*, *E. coli*, lettuce, food safety

## Abstract

Microbial disease outbreaks related to fresh produce consumption, including leafy green vegetables, have increased in recent years. Where contamination occurs, pathogen persistence may represent a risk for consumers’ health. This study analysed the survival of *E. coli* and *L. innocua* on lettuce plants watered with contaminated irrigation water via a single irrigation event and within stored irrigation water. Separate lettuce plants (*Lactuca sativa* var. *capitata*) were irrigated with water spiked with Log_10_ 7 cfu/mL of each of the two strains and survival assessed via direct enumeration, enrichment and qPCR. In parallel, individual 20 L water microcosms were spiked with Log_10_ 7 cfu/mL of the individual strains and sampled at similar time points. Both strains were observed to survive on lettuce plants up to 28 days after inoculation. Direct quantification by culture methods showed a Log_10_ 4 decrease in the concentration of *E. coli* 14 days after inoculation, and a Log_10_ 3 decrease in the concentration of *L. innocua* 10 days after inoculation. *E. coli* was detected in water samples up to 7 days after inoculation and *L. innocua* was detected up to 28 days by direct enumeration. Both strains were recovered from enriched samples up to 28 days after inoculation. These results demonstrate that *E. coli* and *L. innocua* strains are able to persist on lettuce after a single contamination event up until the plants reach a harvestable state. Furthermore, the persistence of *E. coli* and *L. innocua* in water for up to 28 days after inoculation illustrates the potential for multiple plant contamination events from stored irrigation water, emphasising the importance of ensuring that irrigation water is of a high quality.

## 1. Introduction

Leafy green vegetables are an important component of a balanced diet, and their consumption has increased worldwide in recent years [1,2]. This has been linked with a rise in associated microbial disease outbreaks [3,4]. The consumption of contaminated lettuce, in particular, has been linked to several outbreaks [5,6,7,8]. *Escherichia coli* O157 and *Listeria monocytogenes* are two species which have been associated with lettuce contamination, as exemplified by an outbreak linked to the consumption of bagged salad leaves contaminated with *E. coli* O157, which caused 47 illnesses in 2015 in the United Kingdom [9]; another outbreak in the UK caused by the consumption of salad contaminated with *E. coli* O157, which was responsible for 161 illnesses and two deaths [8]; a multi-state outbreak in the United States linked to the consumption of romaine lettuce contaminated with *E. coli* O157, causing 240 infection cases and resulting in five deaths [10]; an outbreak in the US caused by consumption of bagged salads contaminated with *L. monocytogenes* in 2016, which led to 19 infections and one fatality [11]; or an outbreak linked to lettuce contaminated with *L. monocytogenes,* responsible for 99 infections and 15 fatalities in 2011 [12]. *E. coli* O157 is a Shiga toxin producing *E. coli* (STEC) and can cause symptoms of gastrointestinal illness, as well as the more severe haemolytic uremic syndrome (HUS). The most common reservoir of these strains is cattle. *E. coli* O157 isolates have been reported to survive up to 21 months in manure in trials performed in laboratory and field settings [13,14,15,16,17]. *L. monocytogenes*, the causative agent of listeriosis, is found in terrestrial environments, fresh water, salt water, manure and plant material [18,19] and is able to survive in environments with low water activity and grow and multiply under refrigeration [19].

Leafy green vegetables are susceptible to microbial contamination via a number of different pathways at each stage of the farm to fork chain [20]. Contamination during primary production is generally linked to plant contact with contaminated manure [21] or soil [22], the use of contaminated water for irrigation [23,24] or other applications such as product washing or pesticide application. The role of water, specifically, in the contamination of leafy green vegetables has been widely reported in literature [9,25,26,27,28,29]. Water used for irrigation can be obtained from different sources, such as municipal main water supplies, groundwater or surface water collected from rivers, lakes and artificial ponds [30,31]. Fresh water scarcity has also led to increased usage of treated wastewater as an irrigation source [30,32]. The microbiological quality of these water sources, particularly of surface and treated wastewater, is of paramount importance, as microbial pathogens present in the water can be transferred and persist in plant material. Contamination and persistence of *E. coli* and *Listeria* species in lettuce plants after irrigation with contaminated water has been demonstrated in several studies. These studies include work performed in laboratory settings, in warm temperatures and using different crops, such as corn salad, rocket and basil [33], romaine lettuce [34], different varieties of green leafy and romaine lettuce [35] or butterhead lettuce [36]. Field studies have also been conducted, focusing on climate conditions representative of the production of romaine lettuce in the South of Europe, characterised by temperate semi-arid climate [37,38]); production of different varieties of green leafy and romaine lettuce [35] and spinach and green leafy lettuce [39] in North America under humid subtropical climate conditions; production of different herbs in glasshouses in North America in humid subtropical climate conditions [40]; production of romaine lettuce in fields under humid continental climate settings [41]; or romaine lettuce produce under warm-summer Mediterranean climate conditions in North America [42]. However, much more limited information is available about the survival and dissemination of bacterial contaminants in cooler climates. A study has been conducted on the production of butterhead lettuce in greenhouses in temperate maritime climate conditions in Belgium [36], focusing on the survival of *E. coli* and *Salmonella* Thomson, assessed by culture methods. Butterhead lettuce (*Lactuca sativa* var. *capitata*) is a variety typical of Northern European countries, generally produced in a continuous monoculture system in glasshouses with overhead spray irrigation systems [43].

Further to its relevance as a contaminant of fresh produce crops, *E. coli* is of notable interest as an indicator for faecal contamination of water and the potential presence of human enteric pathogens [44]. The detection of generic *E. coli* and other faecal indicator bacteria (FIB) is used to monitor the potential presence of pathogens in fresh water sources, minimising the costs and difficulties associated with testing for individual pathogenic species [45]. *E. coli* and other indicator microorganisms are used, as they should persist in the environment for longer periods than human enteric pathogens and are shed in higher numbers by their animal hosts than human pathogenic microorganisms [46]. Guidelines and regulations in different countries typically refer to the presence/quantification of *E. coli* and faecal or total coliforms as criteria for the microbial quality of irrigation water [31]. In this sense, the presence of *E. coli* in waters used for irrigation and production of fresh crops can be an important indicator for the risk of microbial contamination of crops.

Culture-based approaches and molecular biology methods, such as PCR and/or qPCR, are typically used for the detection of *E. coli* and *L. monocytogenes* species. These methods are also available for detection of *L. innocua* strains, used as a surrogate microorganism to replicate the behaviour of *L. monocytogenes* in settings where the use of the pathogen is not possible. PCR approaches present the advantage of quicker and often same-day results [47], with sample concentrations being the major time-limiting step in the case of water samples. However, these methodologies require skilled operators and a laboratory equipped for sample processing, analysis and interpretation of results [45]. Results obtained with these approaches may be affected by sample interference—specifically, inhibition caused by sample matrix or contaminants—leading to underestimation of targets or even false-negative results [48]. On the other hand, overestimation of target concentration is also a possibility, due to the detection of DNA from non-viable cells [49,50]. Culture methodologies are not affected by these constraints, but usually require longer times to obtain results, with analysis of water samples taking at least 24 to 48h or even more, depending on the method/microorganisms tested. Another issue faced when analysing samples using culture-based approaches is the potential presence of microorganisms in a viable but not culturable (VBNC) state, increasing the risk of false-negative results [51,52].

The objective of this study was to assess the survival of *E. coli* and *L. innocua*, a surrogate microorganism for *L. monocytogenes*, in butterhead lettuce plants produced in typical Irish conditions (temperate oceanic climate) after a single event of overhead spray irrigation with contaminated water using both culture methods and a qPCR approach. The survival of both microorganisms in stored irrigation water for the duration of the trial was also evaluated. Furthermore, this work also aimed to compare the utility of both detection methodologies in produce and water.

## 2. Materials and Methods

### 2.1. Generation of Marked Strains and Inoculum Preparation

Due to biosafety reasons, pathogenic strains could not be used in this study. An environmental isolate of *E. coli,* Lys9 [53,54,55], which possesses survival traits more relevant to environmental conditions than other laboratory adapted *E. coli* strains typically used in persistence studies [53,54], as well as an *L. innocua* ATCC 51742 strain isolated from the plant production environment, were used as representative strains. In order to differentiate the strains used in this trial from the natural microflora present in both the lettuce plants and the potting material, streptomycin-resistant mutants of strains *E. coli* Lys9 and *L. innocua* ATCC 51742 were prepared based on the method of Blackburn and Davies [56]. Each strain was grown in Nutrient Broth (NB-OXOID) at 37 °C for 24 h and then re-inoculated in NB supplemented with increasing concentrations of streptomycin. Mutants resistant to 2 mg/mL of the antibiotic were selected and preserved at −80 °C on cryobeads with 80% glycerol for later use. The growth rate of the mutants was found to be comparable with those of the parent strains by performing growth curves at 37 °C by measuring the optical density at 595 nm for 24 h (data not shown). Three separate inocula of *E. coli* Lys9 StrepR and *L. innocua* ATCC 51742 StrepR were prepared in TBX (TBX-OXOID) and Chromogenic *Listeria* Agar (OCLA-OXOID) supplemented with 0.5 mg/mL of streptomycin, respectively. The *E. coli* strain was incubated at 37 °C for 24 h and the *L. innocua* strain was incubated at 37 °C for 48 h. Individual colonies were picked from each plate, inoculated into NB and incubated at 37 °C for 24 h. Cells were harvested by centrifugation at 7000× *g* for 10 min at 4 °C, washed in Phosphate Buffered Saline (PBS–Sigma Aldrich, Ireland) and resuspended in PBS. The initial concentration of the *E. coli* and *L. innocua* cell suspensions was estimated by plating serial suspension dilutions in TBX and OCLA supplemented with 0.5 mg/mL streptomycin.

### 2.2. Plant Growth Conditions

Seedlings of “butterhead” lettuce (*Lactuca sativa* var. *capitata*) were purchased from a local producer and grown in individual plastic pots (23 cm diameter) with 5 L of a mixture of 20% sterilised loam and 80% peat. The pots were placed in two individual compartments of a glasshouse and irrigated daily throughout the duration of the trial. The trial took place from November to December 2017. The temperatures in the glasshouse rooms were recorded for the duration of the experiment.

### 2.3. Experimental Plot Design

For each strain, four plots of seven by four plants were prepared. These plots were placed adjacent to each other and separated by four extra rows of guard plants. Three plots (plots 1 to 3) were inoculated with contaminated irrigation water, and the remaining plot (plot 4) was irrigated with uninoculated water. Additional plants were prepared to replace plants removed at each sampling point in order to maintain uniform plant growth conditions.

Individual irrigation water microcosms were prepared in 20 L white plastic containers. For each strain, 24 containers were inoculated. A mains water supply was used for filling the microcosms. The water had a pH of 7.5, conductivity of 524 µS/cm and turbidity of <0.1 NTU prior to inoculation. Coliforms, *E. coli* and *L. innocua* were not detected in the water prior to inoculation. Containers were kept in a storage facility away from direct sunlight. The temperature in the storage facility was recorded for the duration of the trial.

### 2.4. Inoculation of Lettuce Plants and Irrigation Water Microcosms

Plants were inoculated with spiked irrigation water when the lettuce seedlings reached the 4 to 6 true leaves state. Irrigation water was inoculated to a final concentration of either Log_10_ 7 cfu/mL of *E. coli* Lys9 StrepR or *L. innocua* ATCC 51742 StrepR. This represents a high level of contamination and a worst-case scenario. Each lettuce plant from plots 1 to 3 was inoculated with 300 mL of contaminated irrigation water via overhead spray irrigation. Lettuce plants from plot 4 were irrigated with uninoculated water in a similar manner. Each individual irrigation water microcosm was inoculated to a final concentration of either Log_10_ 7 cfu/mL of *E. coli* Lys9 StrepR or *L. innocua* ATCC 51742 StrepR. Three groups of eight containers were inoculated with each strain, and eight additional containers with uninoculated irrigation water were prepared. In all cases, there were three biological replicates of each inoculation study.

### 2.5. Sample Collection

Collection of samples was performed using sanitised gloves. Lettuce samples were collected at eight different time points from inoculation until the plants reached a marketable/harvestable stage: immediately after inoculation (t0), after 1 day (t1), 3 days (t3), 7 days (t7), 10 days (t10), 14 days (t14), 21 days (t21) and 28 days (t28). At each sampling time, three randomly selected plants from each plot were removed and immediately transported to the laboratory. Irrigation water microcosms were also sampled periodically after inoculation. At each sampling time, one container from each group was removed and transported to the laboratory for immediate processing.

### 2.6. Detection and Enumeration of Bacteria in Lettuce Plants via Culture

A portion of 25 g of each lettuce plant (inner and outer leaves) was mixed with 225 mL of either Buffered Peptone Water (BPW–OXOID), supplemented with 0.5 mg/mL streptomycin for the detection of *E. coli* Lys9 StrepR, or Half Fraser Broth (HFB–OXOID), supplemented with 0.5 mg/mL streptomycin for detection of *L. innocua* ATCC 51742 StrepR, and then it was homogenised for one min in a stomacher (BagMixer–Interscience). Serial dilutions of the obtained homogenates were plated on TBX supplemented with 0.5 mg/mL streptomycin and incubated at 37 °C for 24 h, or OCLA supplemented with 0.5 mg/mL streptomycin and incubated at 37 °C for 48 h, for direct enumeration of colonies of *E. coli* or *L. innocua*, respectively. In parallel, enrichment of the samples was performed by adding one mL of the obtained homogenate to 10 mL of Minerals Modified Glutamate Broth (MMGB–OXOID), supplemented with 0.5 mg/mL streptomycin and incubating at 37 °C for 24 h for samples inoculated with *E. coli*, or incubation of the suspension of inoculated lettuce plants with HFB at 30 °C for 24 h, followed by adding 100 µL of the incubated suspension to 10 mL of Fraser Broth (FB–Oxoid), supplemented with 0.5 mg/mL streptomycin and incubation at 37 °C for 24 h, for plants inoculated with *L. innocua*. The enriched samples were streaked on TBX supplemented with 0.5 mg/mL streptomycin for confirmation of the presence of *E. coli* Lys9 StrepR, or OCLA supplemented with 0.5 mg/mL streptomycin for confirmation of the presence of *L. innocua* ATCC 51742 StrepR. One ml of the initial plant homogenate was stored at −20 °C for DNA extraction. For enriched samples, a value of 0.5 cfu per 1 mL of concentrated sample was assumed, corresponding to one colony detected over two plates on a 100 dilution, with the limit of quantification calculated based on the volume of the pellet after sample concentration.

### 2.7. Detection and Enumeration of Bacteria in Irrigation Water via Culture

Each water microcosm was concentrated by dead-end ultrafiltration, implementing a methodology developed by the EU project AQUAVALENS. The complete sample (20 L) was filtered through a Rexeed 25A (Asahi-Kasei, Japan) hollow fibre filter with pore size of 30 kDa. The retentate of each sample was back flushed from the filter using 500 mL of a sterile 0.001% Antifoam A, 0.01% Sodium Polyphosphate and 0.01% Tween 80 (Sigma Aldrich) solution. Beef extract (Sigma Aldrich) and a solution of 5X Polyethylene Glycol 8000 and NaCl (Sigma Aldrich) were added to each eluate to a final concentration of 13.33 g/L and 333.33 mL/L, respectively. The mixture was incubated overnight at 4 °C and centrifuged at 10,000× *g* for 30 min at 4 °C. Pellets were resuspended in PBS with 0.001% Antifoam A and 0.01% Tween 80. One mL of each pellet was preserved at −80 °C for DNA extraction. Enumeration of *E. coli* Lys9 StrepR and *L. innocua* ATCC 51742 StrepR and enrichment for the detection of both strains in the concentrated samples were performed as described above for the detection of both species in lettuce samples. For enriched samples, the value of 0.5 cfu per 1 mL of concentrated sample was assumed, corresponding to one colony detected over two plates on a 100 dilution, with the limit of quantification calculated based on the volume of the pellet after sample concentration.

### 2.8. Detection and Enumeration of Bacteria in Lettuce Plants and Irrigation Water via qPCR

For each lettuce sample, DNA was extracted from one mL of cell suspension. For each irrigation water microcosm, DNA was extracted from one mL of the concentrated pellet. Cells were centrifuged at 5000× *g* for 10 min, and DNA extraction was performed using a QIAGEN DNA Mini kit (Qiagen, Hilden, Germany) according to the manufacturer’s instructions. Extracted DNA was preserved at −80 °C until later use.

Detection of *E. coli* Lys9 StrepR and *L. innocua* ATCC 51742 StrepR was performed using a LightCycler 480 SYBR Green I Master Kit (Roche, Penzberg, Germany). qPCR reactions were performed in a Roche 480 LightCycler II (Roche, Penzberg, Germany) following the qPCR kit manufacturer’s instructions: one initial activation step of 5 min at 95 °C, followed by 45 cycles of 10s of denaturation at 95 °C, 10s of annealing at 60 °C and 10s of elongation at 72 °C. For the detection of *E. coli*, the primers uidAF (5′-CAACGAACTGAACTGGCAGA-3′) and uidAR (5′-CATTACGCTGCGATGGAT-3′), targeting the gene *uidA,* were used [57,58]. *L. innocua* was detected targeting the gene *lin02483*, using the primers lipHQF (5′-AACCGGGCCGCTTATGA-3′) and lipHQR (5′-CGAACGCAATTGGTCACG-3′) [59]. Quantification of targets was performed using standard curves built by extracting DNA as described above from known concentrations of each of the tested strains and extrapolating the amplification threshold crossing point values (Ct) of each sample with those obtained from the standard curve.

### 2.9. Live/Dead Staining

In order to assess the viability of *E. coli* and *L. innocua* cells and its potential effect on the obtained results for the persistence of both strains in stored irrigation water (namely the difference between results obtained via culture methods and qPCR), irrigation water microcosms were prepared by inoculating 20 mL of water to a final concentration of Log_10_ 7 cfu/mL of either *E. coli* Lys9 StrepR or *L. innocua* ATCC 51742 StrepR. The inoculated water microcosms were incubated at 6.8 °C in order to simulate the average recorded temperatures for storage of inoculated irrigation water. Immediately after inoculation, and after 35 days, samples inoculated with both strains were collected and stained with the LIVE/DEAD BacLight Bacterial Viability Kit (ThermoFisher Scientific, Waltham, MA, USA). Briefly, for each sample, 5 mL of each sample was harvested and resuspended in 100 µL NaCl 0.85% (Sigma Aldrich, Burlington, MA, USA). Subsequent to this, 10 µL of a 1:1 mix of SYTO^®^9 3.34 mM (excitation/emission 485/498 nm) and propidium iodide 20 mM (excitation/emission 535/617 nm) were added to each sample and incubated in the dark at room temperature for 30 min. Then, 10 µL of each sample was visualised with a Leica DMi8 fluorescence microscope, and images were processed with LAS X software (Leica, Germany).

## 3. Results

### 3.1. Temperature Data

The lettuce spiking trial took place between November and December 2017. The recorded temperatures in both glasshouse compartments ranged from 4.5 to 18 °C, with average temperature values of 11.31 ± 1.51 and 10.96 ± 1.39 °C for the *E. coli* and *L. innocua* compartments, respectively. Recorded temperatures in the irrigation water storage facility ranged from 3 to 11 °C for the duration of the trial, with average temperature values of 6.88 ± 1.92 °C for *E. coli* samples and 6.72 ± 1.26 °C for *L. innocua* samples.

### 3.2. Survival of E. coli and L. innocua in Lettuce Plants

**Figure 1:**
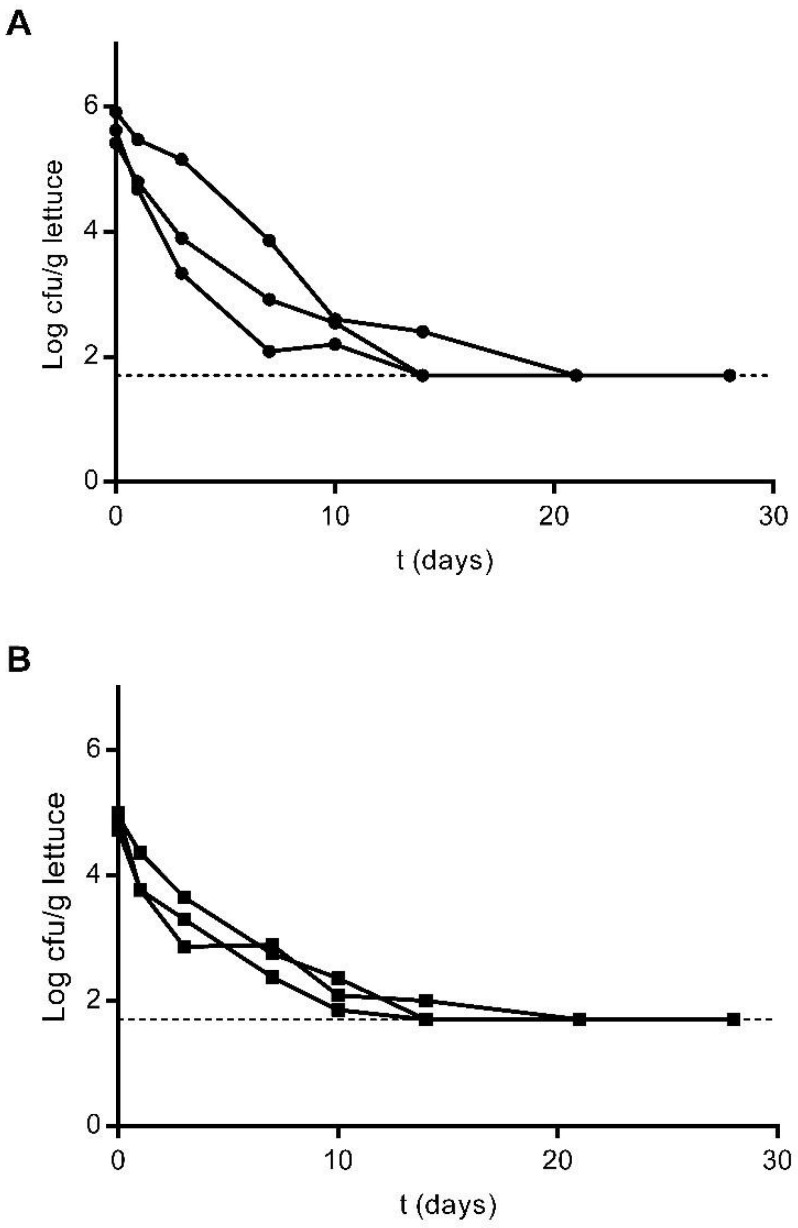
Survival of *E. coli* Lys9 StrepR (**A**) and *L. innocua* ATCC 51472 StrepR (**B**) on lettuce plants after a single contamination event with spiked irrigation water, as determined by culture methodologies up to 28 days after inoculation. Three plants were sampled at each sampling point from each plot and analysed individually. The presented results correspond to direct plating and enrichment for three replicates (individual plots). For enriched samples, the value of 0.5 cfu per 1 mL of suspension was assumed, corresponding to 50 cfu/g of lettuce sample (Log_10_ 1.69). This value was assumed as the quantification limit and is represented with a dashed line.

**Figure 2:**
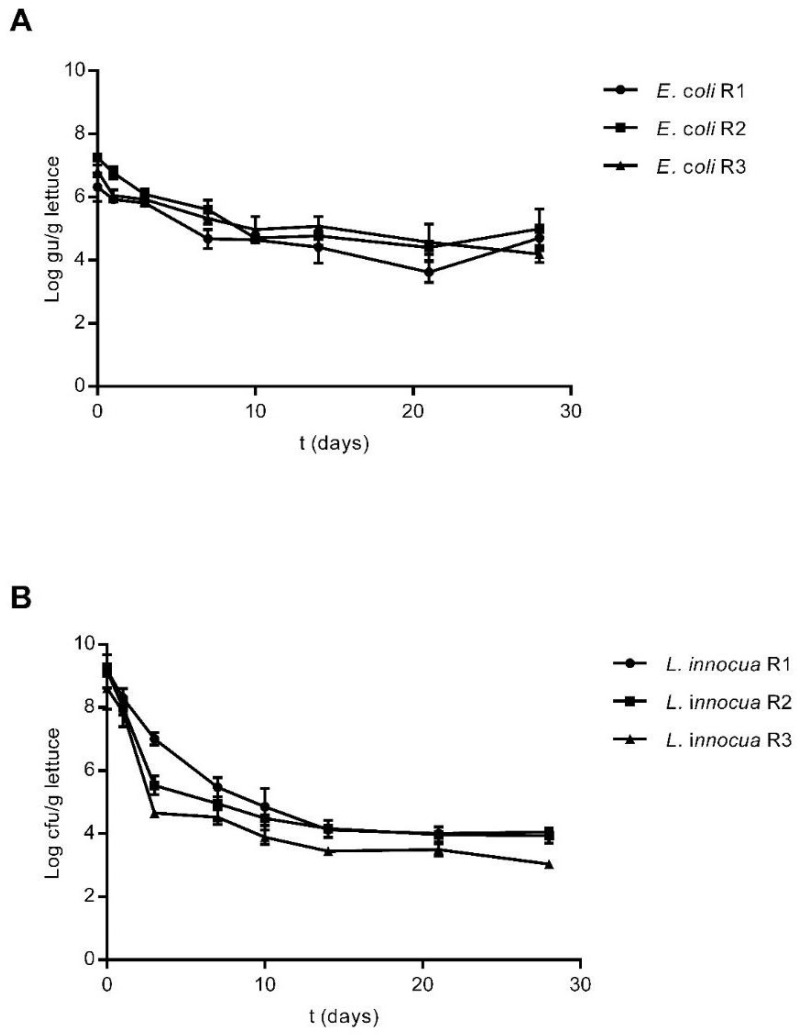
Survival of *E. coli* Lys9 StrepR (**A**) and *L. innocua* ATCC 51472 StrepR (**B**) on lettuce plants after a single contamination event with spiked irrigation water, as determined by qPCR up to 28 days after inoculation. Three plants were sampled at each sampling point from each plot and analysed individually, and the error bars at each time point represent the standard deviation between these technical replicates. Results are presented as Log_10_ gu/g of plant material.

Both *E. coli* and *L. innocua* were detected on plants after a single inoculation event up to 28 days after contamination, when plants reached a harvest-ready state and were detected both by culture methods (Figure 1) and qPCR (Figure 2).

Immediately after inoculation, concentrations of *E. coli* were estimated to be between Log_10_ 5 and Log_10_ 6 cfu/g of plant material by culture methodologies (Figure 1A). Detected levels dropped to Log_10_ 2.09, Log_10_ 2.91 and Log_10_ 3.86 cfu/g of plant material in each of the three inoculated plots after one week, and these values stabilised to a detected minimum of Log_10_ 1.70 cfu/g 14 days after inoculation. On days 21 and 28, the presence of *E. coli* was only observed following enrichment (Figure 1A). A similar trend was observed for the plants inoculated with *L. innocua*. As determined by culture methodologies, the concentration of *L. innocua* immediately after inoculation was estimated to be around Log_10_ 5 cfu/g of plant material (Figure 1B). One week after plant contamination, these values had dropped to below Log_10_ 3 cfu/g of plant material in the three contaminated plots. As observed for the plants contaminated with *E. coli*, after 14 days, lettuce contamination levels had stabilised to Log_10_ 1.70 cfu/g plant material, with *L. innocua* only being detectable by sample enrichment on days 21 and 28 (Figure 1B).

The survival of both strains on lettuce plants was also estimated by qPCR. Estimation of *E. coli* and *L. innocua* levels was carried out by quantification of the *uidA* and *lin02483* genes, respectively. Both gene sequences targeted are present in the respective genomes as a single copy [57,58,59]; therefore, the concentration of the target bacteria in each sample could be estimated based on the number of gene copies detected in each qPCR reaction, with the results being presented as genomic units detected per mL of sample (gu/mL). Both *E. coli* and *L. innocua* were detected up to 28 days after plant inoculation, similarly to what was observed via culture methods (Figure 2).

Upon inoculation of lettuce plants with *E. coli*, detected levels of this strain in the three plots ranged from Log_10_ 6 to Log_10_ 7 gu/g of plant material. After ten days, detected concentrations dropped to values between Log_10_ 4.65 and Log_10_ 4.97 gu/g, with these concentrations remaining stable in each of the three plots up to 28 days after the initial inoculation (Figure 2A). Considering the plants inoculated with *L. innocua*, estimated concentrations immediately after inoculation were higher than those observed for *E. coli*, with detected levels ranging from Log_10_ 8.60 to Log_10_ 9.48 gu/g of lettuce. In two out of three plots, detected levels of *L. innocua* dropped considerably after three days to concentrations of Log_10_ 5.53 and Log_10_ 4.66 gu/g. After ten days, detected concentrations decreased to around Log_10_ 4 in all plots, and these values remained stable up to 28 days after inoculation (Figure 2B).

### 3.3. Survival of E. coli and L. innocua in Stored Irrigation Water

Results for the stored irrigation water microcosms are presented in Table 1. Both *E. coli* and *L. innocua* were detected by culture methods up to 28 days after inoculation in two out of three replicates (Table 1). After inoculation, initial *E. coli* concentrations ranged from Log_10_ 3.74 to Log_10_ 4.60 cfu/mL of irrigation water. One day after inoculation, the concentrations dropped significantly, with observed values of Log_10_ −0.38, Log_10_ 0.29 and Log_10_ 2.31 cfu/mL for each of the individual replicates. In one of the replicates, *E. coli* was only detectable after sample enrichment three days after inoculation, and no bacteria were detected after day 14. In the remaining replicates, *E. coli* was detected by direct enumeration up to three or seven days after inoculation. In both latter replicates, the strain was recovered after sample enrichment up to 28 days after initial inoculation (Table 1). Regarding the survival of *L. innocua* in stored irrigation water samples, the initial detected concentrations ranged from Log_10_ 4.54 to Log_10_ 5.88 cfu/mL of sample. The drop in recovered levels of *Listeria* was not as pronounced as observed with *E. coli*, with concentrations one day after inoculation estimated between Log_10_ 2.75 and Log_10_ 4.84 cfu/L. In one of the replicates, *L. innocua* was only recovered up to 14 days after inoculation. In the remaining two replicates, the strain was recovered up to 28 days after sample inoculation (Table 1).

The detection of both species in irrigation water via qPCR showed that levels of both *E. coli* and *L. innocua* remained stable for the full duration of the trial. Estimated concentrations of *E. coli* ranged from Log_10_ 4.26 to Log_10_ 5.06 gu/mL of sample over 28 days, and *L. innocua* concentrations ranged from Log_10_ 6.07 to Log_10_ 6.85 gu/mL throughout the trial (Table 1).

### 3.4. Live/Dead Staining

**Figure 3:**
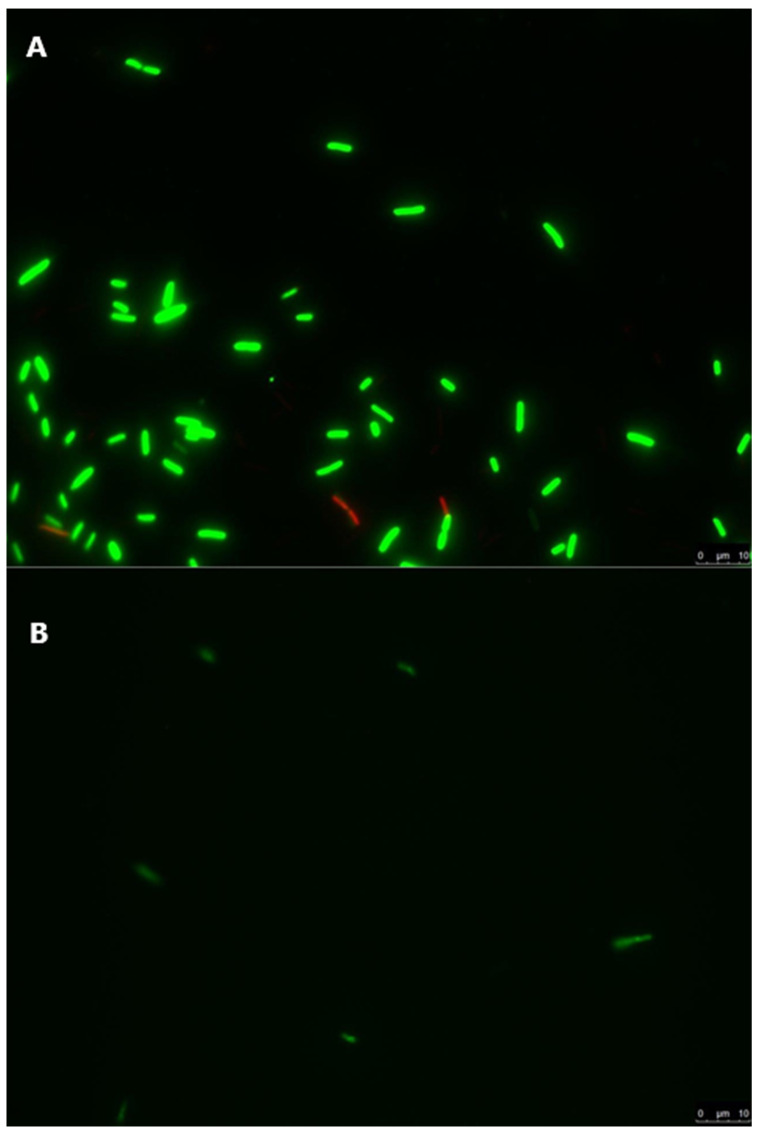
*E. coli* Lys9 StrepR inoculated in stored irrigation water microcosms, labelled with a live/dead staining protocol. Green-labelled cells are live, and red-labelled cells are injured or dead. The scale bar on each picture represents 10 µm. (**A**) *E. coli* Lys9 StrepR immediately after inoculation in stored irrigation water microcosms. (**B**) *E. coli* Lys9 StrepR 35 days after inoculation in stored irrigation water microcosms.

**Figure 4:**
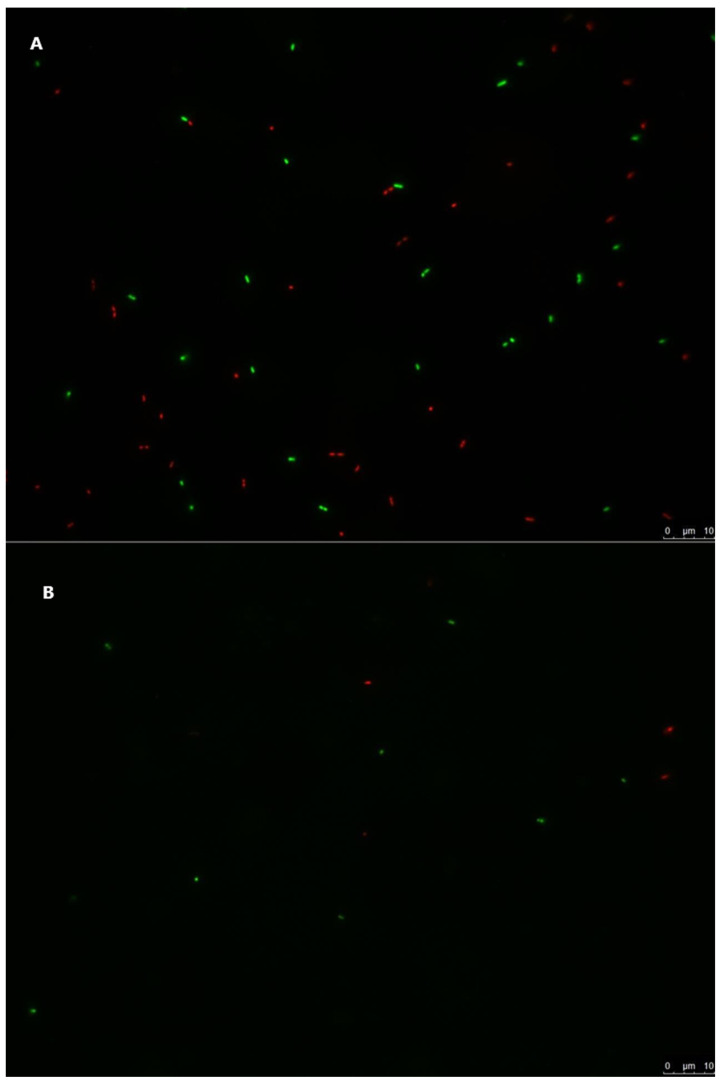
*L. innocua* ATCC 51472 StrepR inoculated in stored irrigation water microcosms, labelled with a live/dead staining protocol. Green-labelled cells are live, and red-labelled cells are injured or dead. The scale bar on each picture represents 10 µm. (**A**) *L. innocua* ATCC 51472 StrepR immediately after inoculation in stored irrigation water microcosms. (**B**) *L. innocua* ATCC 51472 StrepR 35 days after inoculation in stored irrigation water microcosms.

*E. coli* and *L. innocua* cells inoculated in 20mL water microcosms were treated with a live/dead staining protocol, immediately after inoculation or after 35 days, and visualised with a fluorescent microscope. *E. coli* cells collected immediately after inoculation were predominantly alive, as demonstrated by the majority of visible cells being labelled green (Figure 3A). A similar result was observed for *L. innocua* cells collected after inoculation of the water microcosms, although a higher proportion of dead or injured cells were visualised (Figure 4A). The number of visible cells of both strains decreased considerably in the samples collected after 35 days (Figure 3B and Figure 4B), albeit with the majority of observed *E. coli* and *L. innocua* cells being labelled alive. Non-labelled cellular debris was observed in the samples collected 35 days after inoculation for both tested strains (data not shown).

## 4. Discussion

The work presented here demonstrated the ability of both *E. coli* Lys9 and *L. innocua* ATCC 51742 to persist in lettuce plants for long periods of time after a single contamination event with spiked irrigation water in typical winter Irish glasshouse production conditions. The survival of both strains in stored irrigation water was also demonstrated for similar periods of time. Culture- and qPCR-based methodologies were used in this study in order to compare the applicability of each methodology in the proposed setting.

Lettuce plants were inoculated with contaminated overhead spray irrigation water in order to simulate the most typical commercial practices. After a single contamination event, both *E. coli* and *L. innocua* were recovered on lettuce up to 28 days after inoculation, when the plants were ready for harvest and sale, as determined by both culture methods and qPCR (Figure 1 and Figure 2). After inoculation, *E. coli* populations decreased more quickly within the first week, with the remaining cell concentration stabilising within the following two weeks, following a biphasic decay pattern. After 10 days, *E. coli* concentrations fell below the quantification limit of direct detection in two replicates, and the strain was recovered in enriched samples until the conclusion of the trial (Figure 1A). Similar results were observed in plants inoculated with *L. innocua* with a more pronounced decrease in the first week after inoculation, and populations falling below the limit of quantification by direct plating after 14 days (Figure 1B).

Similar results for the survival of both strains on lettuce plants have been demonstrated in previous works simulating different production conditions. A non-toxigenic *E. coli* O157 strain was demonstrated to survive in lettuces grown in greenhouse settings in temperatures similar to those observed in this trial for up to eight days after inoculation [36], and overhead spray irrigation was demonstrated to favour the survival of the strain. Another study performed in greenhouse conditions in warmer temperatures (approximately 21 °C) reported the survival of *E. coli* strains up to 30 days after initial inoculation of the plants with spiked irrigation water, either with single or multiple contamination events [60]. *L. monocytogenes* has been reported to survive in green herbs produced in a glasshouse environment for up to 28 days after inoculation via overhead spray irrigation [40]. Extended survival periods of different strains of *E. coli* and *Listeria* have been demonstrated for other production conditions, such as trials conducted in growth chambers [33,35] or field production conditions [37,38,41,42,61].

The biphasic survival trend observed in this work has been previously reported in different studies with contaminated produce [41,42,62,63,64]. A suggested explanation for this may be that the die-off of bacteria in lettuce plants is mediated by environmental conditions, such as temperature, UV radiation or relative humidity [36,39,63].

Given this, survival of contaminant bacteria will be greater in protected areas of the plants, such as inner leaves, as opposed to older, outer leaves, which will be more exposed to environmental conditions. Moreover, it has been reported that *E. coli* O157 and *Salmonella* have a higher affinity to attach to the bottom and middle part of lettuce leaves [65]. Moreover, due to the morphological characteristics of lettuce plants, there is the potential for retention of irrigation water when delivered via overhead spray. This will facilitate the accumulation of water and potentially pathogenic bacteria in the inner parts of the plant, favouring bacterial survival [32,61]. Another possible scenario that could explain the observed die-off dynamics could be a potential heterogeneity within the inoculated bacteria (specifically heterogeneous bacterial populations in stationary phase) and/or the adaptation of the surviving fraction of the initial inoculum to the plant environment [63]. It has been suggested that the initial die-off rate of different bacteria inoculated on fresh produce in different studies are determined by the conditions in which the inoculum was prepared, held and applied to plants, which in turn influences the ability of the bacteria to adapt to the harsh plant environment [35]. This scenario could account for the observed stabilisation of the levels of *E. coli* and *L. innocua* in the later weeks of the trial. It would be of great interest to phenotypically and genetically compare the original *E. coli* and *L. innocua* strains (i.e., when the inocula for the spiking of the lettuce plants were prepared) with the surviving subpopulations of both strains at 28 days after inoculation of plants in order to assess potential adaptation traits that favour the survival of both strains in the plant environment. In the context of bacterial pathogens, such surviving subpopulations would constitute a concern for lettuce producers and consumers as their persistence increases the likelihood of disease cases and outbreaks among consumers. It is important to note, however, that neither strain used in this study was a human pathogen. Therefore, the observed results in this trial should be interpreted with care, and this should be taken into account when inferring potential risks of contamination of lettuce plants with pathogens.

The survival of both strains in stored irrigation water was also assessed. Both strains were recovered up to 28 days after inoculation (Table 1). *E. coli* was recovered by direct plating up to seven days after the contamination event, and in enriched samples, up to 28 days after inoculation in two of the replicates. In contrast, *L. innocua* was recovered via direct plating up to 21 days after inoculation in two out of three replicates, indicating a slower decrease in detected levels of this strain than what was observed for *E. coli* (Table 1). *E. coli* Lys9 has been previously demonstrated to survive up to 70 days after inoculation in water microcosms [57], with viable cells recovered until the end of the trial. In that study, the reported T_90_ values for the survival of this strain both at 8 and 17 °C were quite high, indicating an ability for this strain to persist in water for long periods of time. However, T_90_ values were lower for unsterilized drinking water than for sterilized water, indicating a faster decay in *E. coli* surviving populations at both temperatures examined [57]. The different experimental set-up of that study (i.e., smaller scale, fixed incubation temperatures as opposed to a varying storage temperature) may explain the differences in the observed results when compared to those obtained in the current study. A study conducted on the survival of several strains of *L. innocua* and *L. monocytogenes* in different water samples demonstrated the survival of the different strains in fresh water stored at 5 °C for up to 40 days, with detected levels of *Listeria* falling below quantification limits 19 days after inoculation [66], a pattern observed in the current study. No significant differences were observed between the survival of the *L. monocytogenes* and *L. innocua* strains tested in that study [66]. Another study on the survival of *L. monocytogenes* in water stored at 4 °C reported survival of the strain up to 100 days after inoculation, and this value was reduced to 50 days when samples were incubated at 20 °C [67]. In the current study, irrigation water was stored at an average temperature of 6.88 °C but ranged between 3 and 11 °C. The variations in temperature may have influenced the survival of both strains in this trial. Other authors have demonstrated the survival of different *E. coli* O157 strains in samples of sterilised groundwater and pond water stored at 4 °C for up to 14 days after inoculation [68], and Abberton and colleagues have pointed the increased survival of *E. coli* Lys9 in sterilised water samples [57], highlighting the risk posed by re-contamination of water after decontamination as this would enhance pathogen survival in the water samples.

A similar survival pattern was observed when estimating the survival of *E. coli* and *L. innocua* in lettuce plants via qPCR, as compared to the results of the culture-based approach. Within the first week after inoculation, there was a marked decrease in detected levels of both strains, followed by a stabilisation of the observed concentrations for the following two weeks of the trial (Figure 2). However, a discrepancy was observed in the results obtained by the two different methodologies. Although the survival pattern observed was similar, the detected concentrations of *E. coli* and *L. innocua* via qPCR were notably higher, especially on days 10 to 28. Estimation of the survival of both strains in stored irrigation water via qPCR also proved to be of limited utility as there was no observable decrease in the detected levels of both *E. coli* and *L. innocua* for the duration of the trial, in contrast to the culture-based results (Table 1), suggesting that there was no degradation of the target DNA in the samples. These results suggest that the qPCR method used may have limited potential as a screening tool for microorganisms present in lettuce and water samples when compared to culture methods, due to the observed overestimation of the concentration of the target strains. One possible reason for the observed discrepancies is the fact that the qPCR method used did not account for cell viability, allowing for the detection of target DNA fragments from non-viable cells [49,50,69]. The presence of cells in a VBNC state could also account for the observed differences, as these cells would still be detectable via qPCR while being non-culturable via conventional methodologies [51,70]. Moreover, irrigation water samples were stored in cold temperatures and protected from sunlight, and these conditions may have contributed to the preservation of the DNA targets in irrigation water, or they may have favoured the tested strains entering a VBNC state [52] as a result of the lack of nutrients and cold storage temperatures. Focusing on the results obtained for water samples, it has been reported that the utilisation of beef extract in the secondary concentration step of the method could lead to detection of *E. coli* DNA in sterilised water samples [71]. This may have also contributed to the stable level of *E. coli* DNA observed in the water samples throughout the duration of the trial.

In order to assess the viability of the tested cells in stored irrigation water, samples were treated with cell viability dyes and observed with fluorescence microscopy [72] (Figure 3 and Figure 4). The observed results demonstrated that some cells of both strains were damaged or dead immediately after inoculation of irrigation water, as a likely result of differences in osmotic pressure and/or the cell harvest process during the preparation of the inocula (Figure 3A and Figure 4A). In samples stored at 6.8 °C for 35 days after inoculation, significantly fewer cells of both strains were observed, and those present were predominantly labelled as live cells (Figure 3B and Figure 4B), with cellular debris present in both samples. This suggests that, over the 35-day storage period, both *E. coli* and *L. innocua* populations decreased in the stored irrigation water, with the majority of dead cells degrading and/or being destroyed. Therefore, the observed levels of both strains in water samples analysed by qPCR (Table 1) would mostly correspond to the detection of non-degraded free DNA from the dead cells of *E. coli* or *L. innocua* preserved in the stored water. The inclusion of this qualitative methodology, or a quantitative method such as PMA-qPCR [51,69]), set up with appropriate calibration curves for the analysis of both the inoculated lettuce and water samples in this trial, in conjunction with culture methods, would likely provide a more accurate estimation of both the persistence and viability of the tested strains. A combination of both approaches would allow for a better estimation of both viable bacteria and bacteria in the VBNC state while also avoiding biases from the qPCR results, such as overestimation of the targeted microorganisms, or to a lesser extent, sample inhibition effects. This information would prove extremely relevant for predicting and managing the risk of produce contamination and reducing the risk of illness for consumers.

## 5. Conclusions

This work has clearly demonstrated the potential for long-term persistence of *E. coli* and *L. innocua* strains on lettuce plants grown in typical Irish glasshouse production facilities after a single inoculation event with contaminated water delivered via overhead spray irrigation. A single inoculation event with overhead spray irrigation water was sufficient for plant contamination, attachment of bacterial cells to plants and persistence of the contaminants. Both tested strains persisted in lettuce plants for up to 28 days after contamination, when plants reached a marketable state, and this poses a potential risk if pathogenic strains exhibit similar survival characteristics. This clearly illustrates the importance of the utilisation of water of good microbiological quality in the production and preparation of fresh produce, given its potential as a vector for microbial contamination. Moreover, both bacterial species proved to be able to persist for extended periods of time in stored irrigation water. This highlights the potential for multiple contamination events in lettuce and other crop production scenarios from storage reservoirs, if contamination occurs. The protection of the irrigation water source and storage facilities is therefore of utmost importance in order to minimise contamination events and ensure the safety of crops. In the context of an increased requirement for the reuse of wastewater of poorer microbiological quality, it emphasises the importance of effective water treatment and appropriate irrigation water placement [30] to avoid crop contamination and to provide product safety assurance. In order to monitor the contamination levels of both the irrigation water and produce samples, an approach including culture methods and a qPCR method that accounts for target viability would be of great value, allowing for the gathering of accurate information regarding the presence and viability of pathogenic microorganisms and providing food producers and risk managers with crucial information to ensure the safety of fresh produce for consumers.

## Figures and Tables

**Table 1:** Survival of *E. coli* Lys9 StrepR and *L. innocua* ATCC 51742 StrepR in 20 L stored irrigation water microcosms up to 28 days after inoculation. Three individual biological replicates were performed for each strain. Survival of each strain was determined via culture methodologies and qPCR after concentration of each full sample.

			*E. coli*						*L. innocua*			
t (Days)	Culture (Log_10_ cfu/mL)			qPCR (Log_10_ gu/mL)			Culture (Log_10_ cfu/mL)			qPCR (Log_10_ gu/mL)		
	Rep 1	Rep 2	Rep 3	Rep 1	Rep 2	Rep 3	Rep 1	Rep 2	Rep 3	Rep 1	Rep 2	Rep 3
0	3.78	3.74	4.60	4.98	5.06	4.58	4.54	5.88	5.77	6.07	6.34	6.14
1	−0.38	0.29	2.31	4.94	4.79	4.84	4.84	3.62	2.75	6.55	6.23	6.61
3	−1.77	−2.35	−1.39	5.02	4.95	4.62	−0.06	−2.43 ^a^	−1.23	6.38	6.36	6.34
7	−2.32 ^a^	0.35	−2.32 ^a^	4.59	4.67	4.73	ND	ND	ND	6.60	6.33	6.39
10	ND ^b^	−2.26 ^a^	−2.26 ^a^	4.68	4.85	4.69	0.75	ND	ND	6.68	6.60	6.66
14	−2.32 ^a^	−2.32 ^a^	−2.32 ^a^	4.71	4.87	4.64	−0.35	ND	ND	6.76	6.35	6.74
21	ND	−2.35 ^a^	−2.35 ^a^	4.26	4.55	4.58	ND	−0.97	−0.06	6.68	6.66	6.53
28	ND	−2.35 ^a^	−2.35 ^a^	4.59	4.68	4.69	ND	−2.35 ^a^	2.01	6.85	6.69	6.69

^a^ The presented culture results correspond to direct sample plating or sample enrichment. For enriched samples, the value of 0.5 cfu/mL of concentrated sample was assumed, with the limit of quantification calculated based on the volume of the pellet after sample concentration. ^b^ ND = not detected.
